# 

**Published:** 1932

**Authors:** 


### The Medical Library of the University of Bristol,

WITH WHICH IS INCORPORATED

**The Library of the Bristol Medico-Chirurgical Society.**

---

*The following donations have been received since the publication of the list in September,* 1932.

November, 1932.

| | | |
|---|---|---|
| British Medical Association (1) | .. .. | 1 volume. |
| V. B. Green-Armytage, Esq. (2) | .. .. | 1 ,, |
| Professor E. W. Hey Groves (3) | .. .. | 7 volumes. |
| Dr. A. Lumière (4) | .. .. .. .. | 1 volume. |
| Dr. T. Milling (5) | .. .. .. .. | 1 ,, |
| Dr. C. Bruce Perry (6) | .. .. .. | 1 ,, |
| Royal College of Surgeons of England (7) | .. | 1 ,, |
| Western Theological Seminary, Illinois, U.S.A. (8) | .. .. .. .. .. .. | 1 ,, |

Unbound periodicals have also been received from Professor E. W. Hey Groves and Dr. Carey F. Coombs.

A collection of pamphlets in fifty-eight volumes on "Head Muscles," together with miscellaneous works were presented by Professor F. H. Edgeworth.

Collections of miscellaneous medical works were received from Dr. A. F. Blagg and Dr. W. Alexander Smith.

### THE ONE HUNDRED AND FORTY-SECOND LIST OF BOOKS.

The figures in round brackets refer to the figures after the names of the donors. The books to which no such figures are attached have either been bought from the Library Fund or received through the *Journal.*

| | | | |
|---|---|---|---|
| **Bastos, M.** .. .. | *Tratado de Patologia Quirurgica General* .. | (3) | 1932 |
| *British Pharmacopœia* .. .. .. | | | 1932 |
| **Campeanu, L.** .. | *Tratamental Chirurgical al Tuberculozei Pulmonare* .. .. .. | (3) | |
| *Clinical Interpretation of Aids to Diagnosis.* Vol. 2.. .. .. .. | | | 1931 |
| **Cope, J.** .. .. .. | *Cancer : Civilization : Degeneration* .. .. .. | | 1932 |
| **Davis, R.** .. .. | *Technique de l'osteosynthèse* .. .. .. | (3) | 1932 |
| **Dietrich, A.** .. .. | *Thrombose ihre Grundlagen und ihre Bedeutung* | (3) | 1932 |

**Douthwaite, A.H.** *Guide to General Practice* .. .. .. .. .. .. 1932
**Drury, E. G. D.** .. *Choosing a Wife and Other Essays* .. .. .. 1932
**Frühwald, V.** .. *Plastic Surgery of the Nose, Ear and Face* .. 1932
**Halford, A. C. F.**.. *Lister Redivivus* .. .. .. .. .. .. .. .. (3) 1928
*International Register of Spas and Mineral Waters* .. .. .. .. .. 1931
**Little, E. M.** .. .. *History of the British Medical Association, 1832–1932* .. .. .. .. .. .. .. .. .. (6) 1932
**Lumière, A.** .. .. *La Vie, la Maladie et la Mort* .. .. (4) 1928
**Oliver, J. R.** .. .. *Pastoral Psychiatry and Mental Health* .. (8) 1932
*Problem of the Out-patient*.. .. .. .. .. .. .. .. .. .. .. .. (1)
*Respice-Prospice, Lewis's,* 1844–1931 .. .. .. .. .. .. .. .. 1931
**Salmon, W.** .. .. *The New London Dispensatory* .. .. (5) 1676
*Société Internationale de Chirurgie, IX Congrès, Madrid* .. .. (3) 1932
**Sorrel, E., and Sorrel-Déjèrine, Y.** *Tuberculose osseuse et ostéo-articulaire* .. .. .. .. .. .. .. .. .. .. (3) 1932
**Stephanus, E. C.** .. *De dissectione partium corporis* .. .. (2) 1545
**Strümpell, A** .. *A Practice of Medicine.* By C. Seyfarth. Translated by C. F. Marshall & C. M. Ottley. 3 vols. 30th Ed. .. .. .. .. .. .. .. .. 1931

TRANSACTIONS, REPORTS, JOURNALS, ETC.

Anales de clinica de hospital Juarez. Tomo 1.. .. .. .. .. .. 1931
Calendar of the Royal College of Surgeons of England, 1932.. (7) 1932
Medical Research Council. Special Report No. 167 .. .. .. .. 1932
Medical Society's Transactions. Vol. LV. .. .. .. .. .. .. .. 1932
Quarterly Cumulative Index Medicus. Vol. 11... .. .. .. .. .. 1932
Saint Bartholomew's Hospital Reports. Vol. LXV... .. .. .. 1932
Saint Thomas Hospital Reports. Vol. LIV. .. .. .. .. .. .. 1932
Surgical Clinics of North America. August, 1932 .. .. .. .. .. 1932

### Local Medical Notes

EXAMINATION RESULTS.

**University of Bristol.**—Students of the University have recently passed the following examinations :—

M.D.—R. M. Norman, C. A. Joll.

M.B., CH.B.—*Supplementary First Examination. Pass :* E. M. Barber, Marjorie R. A. Batten, R. L. J. Derham, P. K. Jenkins, Margaret E. M. Slater.

M.B., CH.B. (*Part I.*).—*Final Examination. Pass:* Grace J. V. Ball, Dorothy E. Barber, A. G. W. Branch, Rosalind M. S. Derham (with distinction in Materia Medica, Pharmacy, Pharmacology, Therapeutics), N. H. Greenberg, G. L. L. Gurney, T. R. V. Gurney, Irene G. Hamilton, J. N. Heales (with distinction in Forensic Medicine and Toxicology), A. J. Matheson, R. A. Mathews, A. C. Molden (with distinction in Materia Medica, Pharmacy, Pharmacology, Therapeutics, and Forensic Medicine and Toxicology), C. H. G. Price (with distinction in Materia Medica, Pharmacy, Pharmacology, Therapeutics and Pathology, and Forensic Medicine and Toxicology), Marion L. Tryon.

M.B., CH.B. (*Part II.*)—*Final Examination. Pass:* R. N. O'D. Burns, J. D. Hughes, J. J. Kempton, J. Ridgway, C. N. Royal, J. P. P. Stock. *In Group II.* (*Completing Examination*)*:* A. C. Price.

B.D.S.—*Supplementary First Examination. Pass:* C. A. Blanden, P. R. Quartley.

L.D.S.—*Second Professional Examination. Pass: In Dental Mechanics and Dental Metallurgy only:* J. G. Clancy.

L.D.S.—*Final Professional Examination. Pass:* R. W. Ellis-Smith, G. N. Minifie, R. L. Royal.

**Conjoint Board**—M.R.C.S., L.R.C.P.—*Pass : In Physics;* Myrtle M. Baxter. *In Elementary Biology:* A. L. T. Beddoe. *In Physiology:* W. H. Hayes. *In Pharmacy and Materia Medica:* S. Roberts. *In Medicine:* G. M. Evans.* *In Pathology:* W. R. Cambridge, D. P. Dewe. *In Medicine and Surgery:* H. E. Pearse.* *In Midwifery:* J. S. Adamson, Aldyth D. Jones, Winifred M. Hill, Violet Fry. *In Surgery;* J. F. H. Eagles, N. C. Coombs.*

L.D.S., R.C.S.—*Final Examination. Pass: Part I.:* C. A. Gear Evans. *Part II.:* F. S. Bromley, J. S. Cochrane, R. H. Green, S. A. McCandlish, P. J. F. Shepherd. H, W. South, W. N. Trays.

* Qualified.

### Index

Adams, A. W.—Ponto-cerebellar tumour, 309.
Albuminuria in pregnancy—
R. S. S. Statham, 219.
J. A. Nixon, 229.
Allison, N. and Ghormley, R. K.—Diagnosis in joint disease (Review), 92.
Anæmia—
Dysphagia with.—E. Watson-Williams, 209.
Pernicious.—G. L. Ormerod, 61.
Appointments—
R. R. Garden.—Assistant Surgeon to Bristol Eye Hospital, 106.
N. H. Kettlewell.—Assistant Lecturer in Orthodontics, University of Bristol, 106.
W. K. Wills.—Sheriff of Bristol, 250.

Bailey, H.—Emergency surgery (Review), 92.
Bailey, H.—A short practice of surgery (Review), 322.
Bellingham-Smith, E. and Feiling, A.—Treatment (Review), 88.
Berry, R. J. A.—Mental deficiency, 177.
Medical education. Pt. 2. (Review), 247.
Birrell, J. A.—Suprarenal virilism, 119.
Brassington, H. W.—Female circumcision amongst Ameru, 237.
Bristol General Hospital, Short history of.—J. O. Symes (Review), 95.
Bristol Medico-Chirurgical Society—
See under Societies.
British Medical Association—
See under Societies.
British Pharmacopœia, 1932—
Letter from G. M. C. re copyright, 168.
(Review.)—A. L. Taylor, 241.
Burdon-Cooper, J., and Roberts, A.—Tungsten-Titanium arc (Review), 91.

Carleton, H. H.—Trigeminal neuralgia, 47.
Cerebello-pontine tumour.—A. W. Adams, 309.
Chambers, E. R.—Common ocular affections, 131.
Charles, J. R.—
Relationship between structure and function, 1.
Suprarenal hypertrophy, 115.
Cheesman, J. E.—Synthetic anatomy (Review), 247.
Choyce, C. C.—A system of surgery (Review), 321.
Christie, H. K.—Grafting skin (Review), 92.
Circumcision, Female, amongst Ameru. —H. W. Brassington, 237.
Coates, V., and Delicati, L.— Rheumatoid arthritis (Review), 90.
Coombs, C. F.—Coronary thrombosis, 277.
Cope, J.—Cancer (Review), 248.
Coronary thrombosis.—C. F. Coombs, 277.
Critchley, Macdonald.—The effects of lightning, 285.
Crookshank, F. G.—Individual diagnosis (Review), 160.
Crowe, H. W.—Handbook of the vaccine treatment of chronic rheumatic diseases (Review), 320.

Deafness.—A. J. M. Wright, 256.
Delmege, J. A.—Health and hygiene in England (Review), 87.
Douthwaite, A. H.—Guide to general practice (Review), 246.
Drury, E. G. D.—Choosing a wife and other essays (Review), 246.
Dysphagia with anæmia.—E. Watson-Williams, 209.

Ear, nose and throat in influenza.—A. J. M. Wright, 123.
Editorial Notes—
A Short History of the Bristol General Hospital, 95.
Branch Meeting of British Medical Association at Taunton, 167.
British Pharmacopœia (1932), 168.
Centenary of the Medical School, 326.
Death of Dr. Carey F. Coombs, 325.
Medical Dinner, 325.
Resignations of Professors Hey Groves and Rayner, 166.
The *Lancet* Commission on Nursing, 97.
The Sheriff of Bristol, 250.
Education, Medical.—C. E. S. Flemming, 199.
Effects of lightning.—Macdonald Critchley, 285.
Ewart, E. D.—Guide to anatomy. (Review), 164.

Z

Eye—
Common ocular affections.—E. R. Chambers, 131.

Fifield, L. R.—Minor surgery (Review), 91.
Flemming, C. E. S.—Medical education, 199.
Forsdike, S.—Textbook of gynæcology. 163.
Frühwald, V.—Plastic surgery of nose, ear and face (Review), 249.

Garrod, L. P., and Hadfield, G.—Recent advances in pathology (Review), 324.
Gordon, R. G.—Insomnia, 147.
Guthrie, A. C.—Research work on pneumococci (Review), 164.
Gye, W. E. and Purdy, W. J.—Cause of cancer (Review), 163.

Hadfield, G., and Garrod, L. P.—Recent advances in pathology (Review), 324.
Handley, W. S.—Genesis of cancer (Review), 93.
Hector, F. J.—Premature infants, 301.
Hillier, W. T.—Formation of animals (Review), 88.
Howe, E G.—Motives and mechanism of the mind (Review), 90.
Hutchison, R.—An index of treatment (Review), 319.
Hutton, I. E.—Hygiene of marriage (Review), 163.
Hypertrophy, Suprarenal.—J. R. Charles, 115.

Infants, Premature.—F. J. Hector, 301.
Influenza, ear, nose and throat in.—A. J. M. Wright, 123.
Insomnia.—R. G. Gordon, 147.

Jaundice, Surgical.—C. A. Moore, 139.

*Lancet* Commission on Nursing. Final Report (Review), 97.
Library Accessions, 103, 172, 253, 332.
Lilley, F. G.—Obituary, 252.
Local Medical Notes—
Examination Results, 105, 174, 254, 333.
Appointments, 106.
Prize Awards, 106.
Long Fox Memorial Lecture, 256.

Maingot, R. H.—The injection treatment of varicose veins, hæmorrhoids, and other conditions (Review), 322.
McCleary, G. F.—National health insurance (Review), 320.
McDowall, R. J. S.—Science of signs and symptoms (Review), 156.
McGregor, A. L.—A synopsis of surgery (Review), 319.
Mental deficiency.—R. J. A. Berry, 177.
Milne Medal in Tropical Medicine.—H. J. H. Spreadbury, 106.
Minty, L. Le Marchant.—The legal and ethical aspects of medical quackery (Review), 321.
Mollison, W. M.—Acute otitis media (Review), 162.
Moore, C. A.—Surgical jaundice, 139.
Myers, C. S.—Human improvability, 31.

Neuralgia, Trigeminal.—H. H. Carleton, 47.
Nixon, J. A.—
Albuminuria in pregnancy, 229.
Provincial medical journals, 312.
Nott, H. W.—Thyroid and manganese treatment (Review), 89.

Obituary—
C. F. Coombs, 326.
F. G. Lilley, 252.
H. L. Ormerod, 251.
Ormerod, G. L.—Pernicious anæmia, 61.
Ormerod, H. L.—Obituary, 251.

Parry, L. A.—Criminal abortion (Review), 159.
Pernicious anæmia.—G. L. Ormerod, 61.
Ponto-cerebellar tumour.—A. W. Adams, 309.
Porter, C.—Sanitary law (Review), 159.
Pregnancy, Albuminuria in—
J. A. Nixon, 229.
R. S. S. Statham, 219.
Premature infants.—F. J. Hector, 301.
Provincial medical journals.—J. A. Nixon, 311.

Resignations—
Professor E. W. Hey Groves, 166.
Professor D. C. Rayner, 166.

Schnek, F.—Non-padded plaster-cast (Review), 161.
Short, A. R.—Index of prognosis (Review), 157.

Societies—
Bristol Medico-Chirurgical Society, 99, 170, 329.
Trigeminal neuralgia.—H. H. Carleton, 99.
Remarks by
J. R. Charles.
G. F. Fawn.
A. R. Short.
C. F. Walters.
E. R. Chambers.
A. E. J. Lister.
A. T. Todd.
R. R. Garden.
A. W. Adams.
A. L. Flemming.
E. W. Hey Groves.
W. P. Kennedy.
Human improvability.—C. S. Myers, 101.
Remarks by
J. R. Charles.
C. F. Walters.
R. G. de M. Rudolf.
F. Bodman.
H. H. Carleton.
Congenital pseudo-hypertrophic muscular paralysis.—G. E. F. Sutton (Case), 101.
Medical treatment of Grave's Disease and toxic goitre.—A. T. Todd, 102.
Remarks by
J. R. Charles.
A. R. Short.
H. H. Carleton.
J. O. Symes.
C. E. K. Herapath.
S. J. H. Griffiths.
R. H. Burnett.
F. Bodman.
N. Neild.
G. E. F. Sutton.
D. A. Alexander.
R. C. Clarke.
Management of ear, nose and throat in influenza.—A. J. M. Wright, 170.
Remarks by
E. Watson-Williams.
J. A. James, junr.
D. A. Alexander.
G. R. Scarff.
C. O. Bodman.
G. Parker.
Some common ocular affections.—E. R. Chambers, 171.
Remarks by
R. R. Garden.
H. Rogers.
C. A. H. Gee.
A. T. F. Rowley.

Societies (*continued*)—
Cerebello-pontine tumour.—A. W. Adams (Case), 172.
Hyperpiesia.—G. E. F. Sutton, 172.
Remarks by
E. R. Chambers.
F. Bodman.
C. B. Perry.
T. Wilson-Smith.
G. Parker.
Some notes on surgical jaundice.—C. A. Moore, 172.
Remarks by
D. Wood.
T. Wilson-Smith.
Electrical injuries.—M.Critchley,331.
British Medical Association (Bath, Bristol and Somerset Branch), 167.

Souttar, H. S.—Radium and cancer (Review), 161.
Statham, R. S. S.—Albuminuria in pregnancy, 219.
Stephens, G. A.—Heart and spleen in health and disease (Review), 158.
Strümpell, A. and Seyfarth, C.—Practice of medicine (Review), 249.
Stubbs, S. G. B. and Bligh, E. W.—Sixty centuries of health and physick (Review), 87.
Suprarenal hypertrophy.—J. R. Charles, 115.
Suprarenal virilism.—J. A. Birrell, 119.
Symes, J. O.—Short history of B.G.H. (Review), 95.

Taylor, A. L.—British Pharmacopœia, 1932 (Review), 241.
Thomson and Miles.—Manual of surgery (Review), 161.
Thrombosis, Coronary.—C. F. Coombs, 277.
Tumour, Ponto-cerebellar.—A. W. Adams, 309.
Turner, A. L.—Diseases of the nose, throat and ear (Review), 323.

Virilism, Suprarenal.—J. A. Birrell, 119.

Wallace, O.—Wayfaring in a splint (Review), 94.
Watkyn-Thomas, F. W.—The principles and practice of otology (Review), 323.
Watson-Williams, E.—Dysphagia with anæmia, 209.
Wells, A. G.—The discharging ear (Review), 162.
Wills, W. K.—Sheriff of Bristol, 250.
Wright, A. J. M.—
Ear, nose and throat in influenza, 123.
The problem of deafness, 256.

J. W. Arrowsmith Ltd. Publishers Bristol

### Publications Received

From Edward Arnold and Co.:

*General Nursing.* By Allan Perry, M.B., M.S., and Dorothy Harvey.

From Baillière, Tindall and Cox:

*A Practice of Medicine.* 3 Vols. 30th Ed. By Adolf Strümpell. Price £5 5s.

*Synopsis of the British Pharmacopœia.* 12th Ed. By H. W. Gadd. Price 2s. 6d.

From Cassell and Company Ltd.:

*System of Surgery.* 3 Vols. 3rd Ed. By C. C. Choyce, C.M.G., C.B.E., B.Sc., M.D.

From The General Medical Council:

*The British Pharmacopœia,* 1932.

From William Heinemann (Medical Books) Ltd.:

*Legal and Ethical Aspects of Medical Quackery.* By L. M. Minty, Ph.D., B.Sc., LL.B. Price 7s. 6d.

From H. K. Lewis and Co. Ltd.:

*National Health Insurance.* By G. F. McCleary, M.D. Price 6s.

*The Breast-fed Baby in General Practice.* By L. G. Housden, M.B., B.S. Price 4s. 6d.

*Common Skin Diseases.* By A. C. Roxburgh, M.A., M.D., B.Ch. Price 18s.

*Short Practice of Surgery.* II. By Hamilton Bailey and R. J. M. Love, M.S. Price 20s.

*Principles and Practice of Otology.* By F. W. Watkyn-Thomas, B.Ch., and A. L. Yates, M.D. Price 25s.

*Extra Pharmacopœia.* Vol. I. 20th Ed. By W. H. Martindale, Ph.D., Ph.Ch. Price 27s. 6d.

*Injection Treatment of Varicose Veins, Hæmorrhoids and Other Conditions.* By Rodney H. Maingot. Price 4s.

*The Principles and Practice of Rectal Surgery.* By William B. Gabriel, M.S. Price 20s.

*The Infant.* By W. T. Pearson, D.M., and A. G. Watkins, M.D., B.Sc. Price 2s. 6d.

*Nervous Disorders in Infancy and Childhood.* By Neill Hobhouse, M.D. Price 8s. 6d.

*Practical Food Inspection.* Vol. I. Meat Inspection. By Charles R. A. Martin. Price 15s.

From Liverpool University Press:

*An Empire Problem.* By D. B. Blacklock, M.D. Price 3s. 6d.

From Oxford University Press:

*Early Diagnosis of the Acute Abdomen.* By Zachary Cope, B.A., M.D., M.S. Price 10s. 6d.

From Williams and Norgate Ltd.:

*The Practice of Birth Control.* By Enid Charles, M.A., Ph.D. Price 10s. 6d.

From John Wright and Sons Ltd.:

*A New Theory of Cancer and Its Treatment.* Part II. By C. F. Marshall, M.Sc., M.D. Price 3s. 6d.

*Elements of Medical Treatment.* 2nd Ed. By Robert Hutchison, M.D. Price 5s.

*Synopsis of Public Health.* By E. W. Caryl Thomas, M.D., B.Sc., D.P.H. Price 21s.

*Synopsis of Surgical Anatomy.* By A. L. McGregor, M.Ch. Price 17s. 6d.

*Diseases of the Nose, Throat and Ear.* 3rd Ed. Edited by A. Logan Turner, M.D., LL.D. Price 20s.

*A Shorter Orthopædic Surgery.* By R. Brooke, M.S. Price 10s. 6d.

*British Journal of Surgery.* Vol. xx. No. 78. Price 12s. 6d.

*Massage and Remedial Exercises.* By N. M. Tidy. Price 15s.

